# Effect of Housing System and Rosemary and Cinnamon Essential Oils on Layers Performance, Egg Quality, Haematological Traits, Blood Chemistry, Immunity, and Antioxidant

**DOI:** 10.3390/ani10020245

**Published:** 2020-02-04

**Authors:** Mahmoud M. Abo Ghanima, Mohamed F. Elsadek, Ayman E. Taha, Mohamed E. Abd El-Hack, Mahmoud Alagawany, Badreldin M. Ahmed, Mona M. Elshafie, Karim El-Sabrout

**Affiliations:** 1 Animal Husbandry and Animal Wealth Development Department, Faculty of Veterinary Medicine, Damanhour University, Damanhour 22511, Egypt; mmyvet2@yahoo.com; 2 Department of Community Health Sciences, College of Applied Medical Sciences, King Saud University, Riyadh 11362, Saudi Arabia; Badreldin222@gmail.com (B.M.A.); melshafie@ksu.edu.sa (M.M.E.); 3 Department of Nutrition and Food Science, Helwan University, Helwan 11795, Egypt; 4 Department of Animal Husbandry and Animal Wealth Development, Faculty of Veterinary Medicine, Alexandria University, Edfina 22758, Egypt; ayman.taha@alexu.edu.eg; 5 Poultry Department, Faculty of Agriculture, Zagazig University, Zagazig 44511, Egypt; dr.mohamed.e.abdalhaq@gmail.com (M.E.A.E.-H.); dr.mahmoud.alagwany@gmail.com (M.A.); 6 Department of Poultry Production, Faculty of Agriculture (El-Shatby), Alexandria University, Alexandria 22758, Egypt

**Keywords:** antioxidant, essential oils, housing system, immunity, ISA brown, production

## Abstract

**Simple Summary:**

The current study aimed to investigate the effects of a housing system, and dietary supplementation of rosemary and cinnamon essential oils on layers performance and egg quality. A factorial arrangement (2 × 3) was performed including two housing systems (floor and cage) and three different types of essential oils (0, 300 mg/kg diet of rosemary and 300 mg/kg diet of cinnamon essential oils) to study their effects on the productive performance, egg quality, immunity, oxidative stress and haematology of laying hens during the production stages. The data suggested that the supplementation of rosemary and cinnamon essential oils in laying hen diet showed significant positive effects on hen performance and egg production. Additionally, the different housing systems did not result in any positive or negative impact on these traits.

**Abstract:**

Housing system and nutrition are non-genetic factors that can improve the well-being of animals to obtain higher quality products. A better understanding of how different housing systems and essential oils can influence the performance of layers is very important at the research and commercial levels. The current study aimed to investigate the effects of a housing system and dietary supplementation of rosemary and cinnamon essential oils on layers’ performance and egg quality. A factorial arrangement (2 × 3) was performed include two housing systems (floor and cage) and three different types of essential oils (0, 300 mg/kg diet of rosemary and 300 mg/kg diet of cinnamon essential oils) to study their effects on the productive performance, egg quality, immunity, oxidative stress and haematology of ISA brown laying hens during the production stages (from 28 to 76 weeks of age). Birds were randomly divided into two groups each comprising of 1500 birds; the first group was moved from the litter to reared laying cages while the second group was floor reared. Each group was randomly divided into three groups, the first was considered as a control group, the second treated with rosemary essential oil, and the third with cinnamon essential oil. The differences in egg production and weight, egg quality, feed intake and conversion, blood picture and chemistry, immunity, and antioxidant parameters between the different housing systems (floor and cage) were not significant at (*p* < 0.05 or 0.01). On the other hand, the egg production and weight, Haugh unit, feed intake and conversion, blood cholesterol, Alanine Aminotransferase (ALT), Aspartate Aminotransferase (AST), urea, Ca, P, immunity, and antioxidant parameters were significantly (*p* < 0.05 or 0.01) better in rosemary and cinnamon groups than in the control group. Furthermore, the results of dietary supplementation with rosemary and cinnamon were very close. Regarding egg production and weight, there were no significant differences due to the interactions. The differences in egg mass among the interactions were also not significant except at 68–76 weeks, where the cage × cinnamon group was the highest. Under the floor rearing system, birds that were fed a diet supplemented with or without essential oils (EOs) consumed more feed than those raised under the cage system. Regarding feed conversion rate (FCR), the differences among the interactions were not significant except at 44–52, 52–60 and 68–76 weeks, where the cage × cinnamon group was the lowest. Excluding glutathione peroxidase (GPx) activity (*p* < 0.001), all immunity and antioxidant indices were not statistically different as a consequence of the interaction among EOs and housing systems. Additionally, the highest levels of phosphorus were observed for layers fed diets enriched with cinnamon oil with the cage or floor system. In conclusion, the data suggested that supplementation of rosemary and cinnamon essential oils in laying hen diet showed significantly positive effects on hen performance and egg production. Cholesterol, liver and kidney functions, immunity, and antioxidant parameters improved with rosemary and cinnamon supplementation when compared to the control. Additionally, the different housing systems did not result in any positive or negative impact on these traits.

## 1. Introduction

Housing systems have always had an impact on animal welfare and performance [1,2,3]. Housing systems, as a non-genetic factor, can improve the well-being of animals to obtain higher quality products. A better understanding of how different housing systems can influence the performance of layers is required. The housing system could influence both the laying hen’s performance and egg quality traits [4]. Englmaierová et al. [4] found that the highest egg production, lowest daily feed consumption, and feed conversion ratio were measured in cages compared to litter. Moreover, higher eggshell, yolk index, and albumen qualities were observed in cages. El-Deek and El-Sabrout [5] concluded that the maintenance of hens in enriched cages and with outdoor access would make it easier for the layers to express their natural behaviour, which has a favourable effect on their welfare and production. Additionally, consumers are recently interested in poultry products originating from alternative housing systems [6], which are natural, organic and have less content of substances that can endanger human health.

In recent decades, plants’ oils have been used routinely in chicken farms for keeping chickens healthy and enhancing their productive performance because they contain active components which exert positive effects on physiological processes and have medicinal effects such as antibacterial, anti-inflammatory, and antioxidant [7,8,9,10]. There is a global trend in restricting the use of antibiotic growth promoters in the animal diet [11] and finding alternative solutions to maintain current animal production efficiency. Essential oils (EOs) have great potential among the alternatives and are generally regarded as safer and residue-free [12]. Due to their preventive and curative properties, species of the Labiatae family have enjoyed a rich tradition of flavoring and pharmaceutical use. Rosemary (*Rosmarinus officinalis*) is an herb that belongs to this family and has been recognized as the plant with the highest antioxidative activity [13]. Rosmarinic acid, camphor, and the antioxidants carnosic acid and carnosol are the most important organic chemicals, which have been already extracted from rosemary [14,15]. The supplementation of rosemary oil (200 mg/kg) in the laying hen’s diet led to a significant improvement in feed conversion and an increase in the Haugh unit (key indicators of internal egg quality) of the egg as well as a larger egg weight [16]. It was also determined that rosemary oil exhibited higher antimicrobial activity than the control (commercial diet) by reducing the *E. coli* concentration in feces. Additionally, using rosemary as a natural antioxidant can decrease plasma total lipids when compared to the control, while LDL-cholesterol and total cholesterol can be insignificantly decreased [7]. Supplementation of 1% rosemary can also improve feed conversion and decreased malonaldehyede (MDA) formation in egg yolk, and has been shown to have a positive impact on oxidative stability of eggshell storage [7]. Moreover, Alagawany and Abd El-Hack [17] concluded that rosemary supplemented up to 6 g/kg diet can be used as an effective feed additive to improve performance, immunity and antioxidant status in laying hens.

On the other hand, cinnamon (*Cinnamomum zeylanicum*) is a common herb and is produced from the bark of the cinnamon tree. Cinnamon herb or its derivatives can serve as a hepatic stimulant by increasing bile secretion, removing toxins, and regulating hydration and can be used as a growth enhancer. Additionally, nutritional aspects of cinnamon powder or its derivatives include positive impacts regarding growth curve, digestion, absorption, activity of gut microbiota, immunity, as well as improved feed utilization and public health of poultry [18]. As a conclusion of Şimşek et al.’s [9] study, working on laying quails, cinnamon essential oil supplementation into a diet with a 200 ppm level increased egg production, eggshell quality, and improved the feed conversion ratio. On the other hand, rosemary supplementation with the same amount did not result in any positive or negative effects on egg production traits while the mixture of both of them had a negative effect on egg weight.

The data concerning the effect of different essential oils (rosemary and cinnamon) under different housing systems (floor and cage) on production, egg quality, immunity, haematology, blood biochemical and antioxidant parameters of layers are rare. Therefore, the objective of this research was to investigate the effects of a housing system, and the supplementation of rosemary and cinnamon essential oils on layers’ performance, haematological traits, blood chemistry, immunity, egg quality, and antioxidants.

## 2. Materials and Methods

All procedures were implemented according to the Local Experimental Animal Care Committee and approved by the ethics of the institutional committee of Damanhour University, Egypt.

### 2.1. Birds and Experimental Design

Three thousand 27-week old ISA brown laying hens were obtained from Al Waha poultry industry (Damo—El Basyounia—El Fayoum—Egypt). A factorial arrangement (2 × 3) was performed, which included two housing systems and three different types of essential oils (0, 300 mg/kg diet of rosemary and 300 mg/kg diet of cinnamon essential oils) to study their effects on the productive performance, egg quality, immunity, oxidative stress and haematology of laying hens during the production stages (from 28 to 76 weeks of age). Birds were randomly divided into two groups each comprising 1500 birds; the first group was moved to laying cages, while the second group was floor reared. Each group was randomly divided into three groups, the first were considered as a control group, the second treated with rosemary essential oil, and the third with cinnamon essential oil. Each group was divided into five equal replicates each of 100 birds. Rosemary essential oil was obtained from Quanao, Shaanxi, China; and cinnamon essential oil was obtained from YiSenYuan, Jiangxi, China (the purity of both oils was 100%). The birds were housed in an open sided farm and each replicate of floor reared layers were housed in a separate pen of about 10 m^2^ space. The pens were separated by nets of 2 m height to avoid group mixing and to avoid interference with air movement, while caged birds in each replicate were housed in separate cages divided into pens, where each pen had dimensions of 40 cm × 50 cm × 40 cm suitable for five birds. The cage consisted of two levels, with each level containing 10 pens (5 on each side).

### 2.2. Dietary Treatments

Composition of basal diet and their calculated analysis is presented in Table 1. The hens were fed diets in mash form during the experiment (28–76 weeks). The diets were formulated to meet or exceed NRC [19] recommendations.

### 2.3. Estimation of Laying Performance Parameters and Egg Quality

Hen-day egg production (HDEP), feed consumption and egg weight was recorded daily on a replicate basis. Feed intake was calculated by subtracting the remaining feed daily from the offered feed. Feed conversion ratio was calculated as grams of feed intake per gram of egg mass produced. Average egg mass (per hen per day in grams) = % HDEP × Average egg weight in grams. The parameters relative to egg quality were evaluated at 72 weeks of age. Fifteen eggs were randomly collected per treatment to determine these parameters. The collected eggs were weighed using a digital balance. On breaking, the egg contents were poured. The Haugh unit (HU) was measured for the internal quality of the eggs [20]. The height, correlated with the weight, determined the HU. The higher the number, the better the quality of the egg (fresher, higher quality eggs have thicker whites). Eggshell, albumin, and yolk percentages were also measured. Eggshell thickness (without the shell membrane) was measured from the middle part of the egg using a micrometer. Yolk index was calculated by formula: Yolk height/Yolk width × 100; while the egg index was calculating using the following formula: (Egg width/egg length) × 100.

### 2.4. Estimation of Blood Haematological and Biochemical Parameters

Blood samples (*n* = 25) were collected from each group as five samples from each replicate from the wing vein at 56 weeks of age. After collecting the blood samples, the tubes were left in the slope position until serum samples were separated through centrifugation at 3000 rpm for 15 min. Red blood cells (RBCs) and white blood cells (WBCs) counts were determined according to Stoskopf [21] using haemocytometer. Blood hemoglobin (HB) was assessed by cyanomta-hemoglobin method [22]. Packed cell volume (PCV) was carried out by using microhaematocrit capillary tubes centrifuged at 12,000 rpm for 5 min. The reading was made with the aid of a microhaematocrit reader and expressed as the volume of erythrocytes per 100 cm^3^ according to Blaxhall and Daisley [23]. Differential leucocytic counts were defined using a blood film that was prepared according to the method described by Lucky [24]. Ten drops from May-Grunwald stain stock solution on a dry, unfixed smear were added to an equal amount of distilled water, then mixed and left for 1 min for staining. The dye was decanted without rinsing. Diluted Giemsa’s solution (10 drops of the dye were added to 10 mL of distilled water) was poured over the film as a counter stain and left for 20 min, then rinsed in water current and examined by oil immersion lens. The percentage and absolute value for each of the type of cells were calculated according to Schalm et al. [25]. The sera were collected and preserved in a deep freezer at (−20 °C) until the time of analysis. All the following studied parameters were calorimetrically evaluated. Estimation of blood cholesterol content was determined by cholesterol kit of Bio-diagnostic according to Richmond [26] and Allain et al. [27]. Total protein was determined by kits of Bio-diagnostic according to the method of Gornal et al. [28]. Alanine Aminotransferase (ALT) was determined by the ALT kit of Bio-diagnostic according to the method of Reitman and Frankel [29], while Aspartate Aminotransferase (AST) was determined by the AST kit of Bio-diagnostic according to the method of Retiman and Frankel [29]. Creatinine was defined according to the method of Bartles et al. [30], while urea was defined according to the method of Fawcett and Scott [31]. Calcium (Ca) and Phosphorus (P) were measured spectrophotometrically by using commercial kits (Spectrum chemical company, PO. Box 30, Obour City—Cairo, Egypt).

#### 2.4.1. Estimation of Malondialdehyde, Glutathione Peroxidase and Super Oxide Dismutase

Estimation of blood Malondialdehyde (MDA) concentration was measured by the method of Jo and Ahn [32]. Determination of Glutathione peroxidase (GPx) activity measured using the Paglia and Valentine [33] spectrophotometric method based on the Northwest Life Science Specialties (NWLSS™) Glutathione peroxidase assay kits protocol NWK-GPX01. Determination of Super Oxide Dismutase (SOD) activity was assessed using the NWLSS™ Superoxide dismutase activity assay, which provided a simple, rate method for determining SOD activity. This method is based on monitoring the auto-oxidation rate of haematoxylin as originally described by Martin Jr. et al. [34].

#### 2.4.2. Estimation of Phagocytic Index, Phagocytic Activity and Cellular Immunity

Blood and serum samples were collected at 56 days of age (five samples per replicate and total 25 samples per each group) as mentioned above according to Stott and Fellah [35] and used for the determination of Phagocytic activity and phagocytic index was determined according to Kawahara et al. [36]. Fifty micrograms of Candida albicans culture was added to 1 mL of citrated blood from each sample and incubated in a water bath at 25 °C for five hours, and then blood smears from each tube were stained with Giemsa stain. Phagocytosis was estimated by determining the proportion of macrophages, which contained intracellular yeast cells in a random count of 300 macrophages and expressed as percentage of phagocytic activity (PA). The number of phagocytized organisms was counted in the phagocytic cells and called the phagocytic index (PI).

Phagocytic activity (PA) = Percentage of phagocytic cells containing yeast cells.

Phagocytic index (PI) = Number of yeast cells phagocytized/Number of phagocytic cells.

### 2.5. Statistical Analysis

Data were analyzed by the statistical analysis system SAS [37]. A 2 × 3 factorial design was used to analyze data of performance as a response to two housing systems and three different types of essential oils. Differences among means were detected using two-way analysis of variance (ANOVA). The differences among means were determined using the Duncan test (*p* < 0.05). The model used was:

Y_ij_ = μ + D_i_ + A_j_ + DA_ij_ + e_ij_

where: Y_ij_ = an observation, μ = the overall mean, D_i_ = fixed effect of housing system, A_j_ = fixed effect of essential oils, DA_ij_ = fixed effect of interaction between housing system and essential oils and e_ij_ = random error associated to each observation.

## 3. Results and Discussion

The effects of different housing systems, essential oils supplementations and their interaction on the egg production of laying hens is shown in Table 2. The differences in egg production percentages between the different housing systems (floor and cage) were not significant at *p* < 0.05 or 0.01. This finding was in agreement with the results of Zita et al. [38]. They indicated no effect of housing system on the egg production of hens. Egg production percentages were significantly (*p* < 0.05 or 0.01) higher in the rosemary and cinnamon groups than in the control group, whereas there were no significant interactions. Şimşek et al. [9] found that cinnamon supplementation helped to increase the egg production. Moreover, Ding et al. [39] revealed that hen-day egg production was significantly improved (*p* < 0.05) at 58 to 61 weeks with the diet supplemented with essential oils Enviva commercial product (50, 100, and 150 mg/kg) including thymol 13.5% and cinnamaldehyde 4.5%.

The effects of different housing systems, essential oils supplementations and their interaction on egg weight of laying hens are shown in Table 3. The differences in egg weight between the different housing systems (floor and cage) were not significant at (*p* < 0.05 or 0.01). This finding was also in agreement with the results of Zita et al. [38]. The egg weights were significantly (*p* < 0.05 or 0.01) higher in rosemary and cinnamon groups than in the control group at 28–36 and 52–60 weeks while there were no significant interactions. The highest egg weight was found in the rosemary group at 44–52 weeks and 60–68 weeks. In agreement, Şimşek et al. [9] reported that the highest egg weight in quail was found in the rosemary group. On the other hand, Alagawany and Abd El-Hack [17] reported that there were no differences in egg weight due to adding rosemary to laying hens. Furthermore, Ding et al. [39] and Cufadar [40] reported that egg weight was not affected by the diet supplemented with essential oils.

The effects of different housing systems, essential oils supplementations and their interaction on egg mass of laying hens are shown in Table 4. The differences in egg mass between the different housing systems (floor and cage) were not significant at (*p* < 0.05 or 0.01). The egg masses were significantly (*p* < 0.05 or 0.01) higher in rosemary and cinnamon groups than in the control group at 28–36, 44–52, 52–60, 60–68 and 68–76 weeks, while the differences among the interactions were not significant except at 68–76 weeks, and the cage × cinnamon group was the highest. In agreement, Alagawany and Abd El-Hack [17] reported that egg mass linearly increased with rosemary supplementation, while Cufadar [40] indicated that there were no differences in egg mass due to the addition of rosemary in laying hen diet.

The effects of different housing systems, essential oils supplementations and their interaction on feed intake of laying hens are shown in Table 5. The differences in feed intake between the different housing systems (floor and cage) were not significant at *p* < 0.05 or 0.01. The feed intakes were significantly (*p* < 0.05 or 0.01) lower in the rosemary and cinnamon groups than in the control group at 60–68 and 68–76 weeks. Apart from feed intake at 44–52 weeks, there were no significant differences due to the interaction effect between EOs and housing system. Under the floor system, birds fed diet supplemented with or without EOs consumed more feed (127.40 to 128.62 g) than those raised under the cage system (119.50 to 124.60 g). Feed intake was found to be similar between the rosemary and cinnamon groups as mentioned before by Şimşek et al. [9]. Although, essential oils are perceived as growth promoters in poultry diets [41], recent studies on poultry feeding intakes [42,43] have indicated that dietary incorporation of essential oils did not significantly affect the bird feed intake or they could decrease it insignificantly. However, there is a possible explanation for the reduced intake of feed is the irritating scent of essential oils, which makes the diet unpleasant to birds.

The effects of different housing systems, essential oils supplementations and their interaction on feed conversion of laying hens are shown in Table 6. The differences in feed conversion between the different housing systems (floor and cage) were not significant at *p* < 0.05 or 0.01. The feed conversions were significantly (*p* < 0.05 or 0.01) lower in the rosemary and cinnamon groups than in the control group at 28–36, 36–44, 52–60, 60–68 and 68–76 weeks, while the differences among the interactions were not significant at (*p* < 0.05 or 0.01) except at 44–52, 52–60, and 68–76 weeks, with the lowest rates being observed for layers fed diets enriched with EOs in the cage. The cage × rosemary group achieved the best values of FCR at 44–52 and 52–60 weeks; while at 68–76 weeks, the best value was recorded by the cage × cinnamon group. It can be concluded that the interactions among cage and EOs system achieved the good results for the FCR in comparison with the floor system. Şimşek et al. [9] found that the best feed conversion was obtained in the cinnamon group. Ding et al. [39] reported that the hen feed conversion ratio was significantly improved (*p* < 0.05) at 58 to 61 weeks with the diet supplemented with essential oils. On the other hand, Alagawany and Abd El-Hack [17] reported that there were no differences in the feed consumption and feed conversion ratio due to adding rosemary to laying hens. However, there are two acceptable mechanisms to understand the effect of these essential oils. The first one considers the promotion of digestive enzyme secretion, and the second deals with the gut microflora ecosystem stabilization, leading to enhanced utilization of food and decreased exposure to growth-depressing disorders that could be related to the metabolism and the digestion processes [44,45,46]. Several chicken studies have documented positive effects of essential oils on the digestive enzyme (pancreatic α-amylase and intestinal maltase) secretion and intestinal mucosa [47,48]. In broilers, the ileal activity of trypsin and chymotrypsin was significantly increased in the thymol group at day 21 compared with the control group [49]. In the in vitro study, Mathlouthi et al. [50] stated that rosemary essential oils had different antimicrobial impacts against pathogenic microbes however, had the same effect on avilamycin as a growth promoter when added to broiler rations. Furthermore, the latter authors found that in vivo growth promotion effects were due to the alterations in the gut microbiota rather than antimicrobial activities against a single bacterial species and genus. Decreased microbes in the gastrointestinal tract may enhance the proliferation ability of epithelial cells and thus improve intestinal absorptive capacity [51]. These effects have been confirmed by increased nutrient digestibility, however, this has not resulted in improved growth performance [52,53].

The effects of different housing systems, essential oils supplementations and their interaction on egg quality of laying hens are shown in Table 7. The differences in egg quality traits between the different housing systems (floor and cage) were not significant at *p* < 0.05 or 0.01. In contrast, Tumova and Ebeid [54] found that the egg quality characteristics were better in eggs produced in cages when compared to alternative systems, and eggs produced from cage systems had higher values of Haugh units, albumen and yolk indices. Furthermore, higher eggshell and albumen qualities were observed in conventional cages by Englmaierová et al. [4]. The differences between the study’s results could be due to differences in the strain used or the environmental conditions surrounding it (such as cages indoors or outdoors), as well as differences in the cages design. However, the differences in shell thickness among the different essential oil groups (including the control group) were also not significant (*p* < 0.05 or 0.01). On the other hand, Cufadar [40] reported that eggshell thickness was significantly increased with rosemary essential oil supplementation (250 mg/kg) in the diet of NOVOgen White laying hens. In general, Ding et al. [39] reported that eggshell thickness was significantly increased at 65 weeks with the diet supplemented with essential oils. Haugh unit scores were higher in rosemary and cinnamon groups than in the control group, while the control group was the highest in yolk index among the interactions at *p* < 0.05 or 0.01. It means that rosemary and cinnamon supplementation (0.3 g/kg) decreased the egg yolk index for Isa Brown laying hens. On the other hand, Alagawany and Abd El-Hack [17] found that adding rosemary (up 6 g/kg) to Hi-sex Brown laying hen’ diets resulted in a linear increase in yolk percent and yolk-to-albumen ratio. While, Botsoglou et al. [55] indicated that diets supplemented with rosemary oil (5 g/kg) for Lohmann laying hens had no effects on the yolk index neither Haugh units. These conflicting results may be due to the different supplementation ratio or/and the strain used. Furthermore, Ding et al. [39] reported that Haugh units, generally, were not affected by the diet supplemented with essential oils.

The effects of different housing systems, essential oils supplementations and their interaction on the blood picture (haematological traits) of laying hens are shown in Table 8. The differences in blood picture between the different housing systems (floor and cage) and among the different essential oils groups were not significant at *p* < 0.05 or 0.01 except in some differential WBCs (basophils; lymphocytes and monocytes %).

The effects of different housing systems, essential oils supplementations and their interaction on immunity and antioxidant parameters of laying hens are shown in Table 9. The differences in immunity and antioxidant parameters between the different housing systems (floor and cage) were not significant at *p* < 0.05 or 0.01 while among the different essential oils groups, they were significant in ND, AI H5, AI H9, MDA, and SOD. Excluding GPx activity (*p* < 0.001), all immunity and antioxidant indices were not statistically different as a consequence of the interaction among housing systems and EOs. The interaction between floor and basal diet gave the highest (26.25 U/gHb) activity of GPx in comparison with the other interactions. However, the lowest value (15.00 U/gHb) was found in birds fed with a control diet with the cage system.

In general, some designs of housing systems can cause some stress on hens. This stress can play an effective role in the bird’s immune system resulting in failure of vaccination or increased disease during production [56,57]. However, essential oils are a total of volatile constituents, and therefore the effects of essential oils could be a complete product of all components and their interactions. Two or three components can account for up to 85% of the total mixture, and thereby contribute to the primary property of the essential oil mixture [58]. For example, phenols (thymol and carvacrol) account for more than 70% of plant essential oils and are primarily responsible for their antibacterial and antioxidant functions. Rosemary has been recognized as the plant with the highest anti-oxidative activity [13]. On the other hand, cinnamon or its oil play an important role in improving the growth, production, digestion, absorption, activity of gut microbiota, immunity, as well as feed utilization and public health of poultry [18].

Through supplementing essential oils, a bird’s anti-oxidative stability can be enhanced [12]. Placha et al. [59] found that malondialdehyde concentration in the liver and kidney was significantly reduced by the supplementation of essential oils to bird diet. Moreover, Lee et al. [60] and Khan et al. [61] reported that thyme essential oil had a significant effect on avian-derived products (meat and eggs) retarding oxidant degradation. Antioxidant activity could be derived from the phenolic OH group, which acts as a hydrogen donor interacting with peroxy radicals during the initial process of lipid oxidation and thereby inhibiting the formation of hydroxyl peroxide [60]. The antioxidant effect differs from plant essential oil to others depending on the amount of total phenols in it. With its successful antioxidant activities in broiler meat, rosemary has been identified [62,63].

The effects of different housing systems, essential oils supplementations and their interaction on the blood chemistry of laying hens are shown in Table 10.

The differences in blood chemistry including the cholesterol between the different housing systems (floor and cage) were not significant at *p* < 0.05 or 0.01. Contrary to this, Zita et al. [38] found that the blood cholesterol level was higher in birds raised in cages than on litter. However, the cholesterol, ALT, AST, and urea levels were significantly (*p* < 0.05 or 0.01) lower in rosemary and cinnamon groups than in the control group. The effect of interaction between dietary the interaction among EOs and housing systems was not significant (*p* < 0.05) on blood chemistry tests except for the blood phosphorus content, the highest levels were observed for birds fed diets enriched with cinnamon oil with a cage or floor system. Alagawany and Abd El-Hack [17] found that serum constituents were not significantly influenced by rosemary supplementation, except for urea and cholesterol. Moreover, Torki et al. [64] reported that birds given rosemary exhibited lower serum cholesterol and triglycerides concentration. The Ca and P were higher in rosemary and cinnamon groups than in the control group.

## 4. Conclusions

The supplementation of rosemary and cinnamon essential oils had great impacts on egg production and weight, some egg quality traits, feed intake and conversion, some haematological traits and blood chemistry, immunity, and antioxidant parameters. On the other hand, the different housing systems did not result in any positive or negative impact on these studied traits. However, the cage × cinnamon group was the highest in egg mass, and the lowest in FCR. Therefore, we have recommended the usage of rosemary or cinnamon essential oils at 300 mg/kg in layer’s diet to improve its productive performance and egg production.

## Figures and Tables

**Table 1:** Ingredients and calculated analysis of layer basal diet.

Item	%
Ingredients	
Yellow corn	61.23
Soybean meal (44% protein)	19.02
Corn gluten meal (60% protein)	7.02
Vitamins and minerals premix *	0.30
Wheat bran	0.46
Calcium carbonate	1.36
Di-calcium phosphate	8.96
DL-methionine	0.05
NaCl	0.40
Lysine	1.20
Chemical analysis (%) **	
Crude protein	18.01
Metabolic energy (Kcal/kg)	2800
Crude fiber	2.85
Calcium	3.81
Phosphorus	0.63

* Each diet was supplied with 3 kg/ton Vitamins and Minerals Mix (commercial source B. p. Max) Each 3 kg contains, Vit. A 10,000,000 MIU, Vit. D 2,000,000 MIU, Vit. E 10,000 mg, Vit. K3 1000 mg, Vit. B1 1000 mg, Vit. B2 5000 mg, Vit. B6 1500 mg, Biotin 50 mg, BHT 10,000 mg, Pantothenic 10,000 mg, folic acid 1000 mg, Nicotinic acid 30,000 mg Mn 60 g, Zinc 50 g, Fe 30 g, Cu 4 g, I 3 g, Selenium 0.1 g and Co 0.1 g. ** The diets were formulated to meet or exceed NRC [19] recommendations.

**Table 2:** Egg production of laying hens as affected by different housing systems, essential oils and their interaction during the experiment.

Items	Egg Production % During					
	28–36 week	36–44 week	44–52 week	52–60 week	60–68 week	68–76 week
Housing system						
Cage	87.86	88.89	85.12	79.76	71.99	62.60
Floor	85.11	85.36	81.20	75.44	68.45	58.83
Essential oils (EOs) ^1^						
0	83.87 ^b^	84.68 ^b^	80.18 ^b^	73.06 ^b^	65.56 ^b^	56.00 ^b^
Rosemary EO	88.30 ^a^	88.95 ^a^	85.40 ^a^	80.15 ^a^	72.25 ^a^	63.00 ^a^
Cinnamon EO	87.30 ^a^	87.75 ^a^	83.90 ^a^	79.60 ^a^	72.85 ^a^	63.15 ^a^
SEM ^2^	0.705	1.142	1.412	1.373	1.287	0.927
Probability						
Housing system	<0.001	0.001	0.004	0.001	0.004	<0.001
EOs	<0.001	0.002	0.007	<0.001	<0.001	<0.001

Means in the same column within each classification bearing different letters are significantly different. (*p* < 0.05 or 0.01). ^1^ EOs: Essential oils, EO: Essential oil. ^2^ SEM: standard error mean.

**Table 3:** Egg weight of laying hens as affected by different housing systems, essential oils and their interaction during the experiment.

Items	Egg Weight (g) During					
	28–36 week	36–44 week	44–52 week	52–60 week	60–68 week	68–76 week
Housing system						
Cage	45.52	51.03	55.27	57.93	59.24	60.35
Floor	43.90	50.69	55.15	57.11	58.65	59.82
Essential oils (EOs) ^1^						
0	41.93 ^b^	49.93	54.75 ^b^	56.12 ^b^	57.93 ^b^	59.06
Rosemary EO	46.00 ^a^	51.95	56.80 ^a^	58.70 ^a^	59.70 ^a^	60.80
Cinnamon EO	46.20 ^a^	50.70	54.10 ^b^	57.75 ^a^	59.20 ^ab^	60.40
SEM ^2^	0.827	0.529	0.529	0.561	0.502	0.722
Probability						
Housing system	0.015	0.632	0.844	0.083	0.180	0.349
EOs	<0.001	0.086	0.002	0.001	0.010	0.050

Means in the same column within each classification bearing different letters are significantly different. (*p* < 0.05 or 0.01). ^1^ EOs: Essential oils, EO: Essential oil. ^2^ SEM: standard error mean.

**Table 4:** Egg mass of laying hens as affected by different housing systems, essential oils and their interaction during the experiment.

Items		Egg Mass (g) During					
		28–36 week	36–44 week	44–52 week	52–60 week	60–68 week	68–76 week
Housing system							
	Cage	43.60	46.26	47.14	44.69	40.13	18.35
	Floor	40.61	44.74	43.90	41.83	37.87	16.94
Essential oils (EOs) ^1^							
	0	38.56 ^b^	44.31 ^b^	42.15 ^b^	39.66 ^b^	35.68 ^b^	15.89 ^b^
	Rosemary EO	44.35 ^a^	47.32 ^a^	48.32 ^a^	45.30 ^a^	40.52 ^a^	18.69 ^a^
	Cinnamon EO	43.41^a^	44.86 ^b^	46.08 ^a^	44.82 ^a^	40.80 ^a^	18.36 ^a^
Housing × EOs							
Cage	0	39.76	45.78	44.60	41.31	36.69	16.88^c^
	Rosemary EO	45.69	47.36	49.52	46.04	41.20	18.78 ^ab^
	Cinnamon EO	45.37	45.64	47.30	46.72	42.49	19.40 ^a^
Floor	0	37.35	42.85	39.71	38.02	34.66	14.90 ^d^
	Rosemary EO	43.02	47.27	47.12	44.56	39.85	18.59 ^ab^
	Cinnamon EO	41.46	44.09	44.87	42.92	39.10	17.32 ^b^
SEM ^2^		0.829	0.856	0.980	0.837	0.760	0.300
Probability							
Housing system		<0.001	0.048	0.001	0.001	0.002	<0.001
EOs		<0.001	<0.006	<0.001	<0.001	<0.001	<0.001
Housing × EOs		0.650	0.310	0.416	0.368	0.407	0.008

Means in the same column within each classification bearing different letters are significantly different. (*p* < 0.05 or 0.01). ^1^ EOs: Essential oils, EO: Essential oil. ^2^ SEM: standard error mean.

**Table 5:** Feed intake (g) of laying hens as affected by different housing systems, essential oils and their interaction during the experiment.

Items		Feed Intake (g) During					
		28–36 week	36–44 week	44–52 week	52–60 week	60–68 week	68–76 week
Housing system							
	Cage	88.89	108.58	122.30	126.83	121.06	116.04
	Floor	97.15	114.41	127.84	136.41	129.44	117.80
Essential oils (EOs) ^1^							
	0	93.81	112.75	124.06	132.37	127.31 ^a^	117.87 ^a^
	Rosemary EO	92.60	110.95	126.00	130.85	123.95 ^b^	116.30 ^b^
	Cinnamon EO	92.65	110.80	125.15	131.65	124.50 ^b^	116.60 ^ab^
Housing × EOs							
Cage	0	89.87	110.75	119.50 ^d^	127.00	122.50	116.62
	Rosemary EO	88.60	107.70	124.60 ^b^	127.00	120.30	116.20
	Cinnamon EO	88.20	107.30	122.80 ^c^	126.50	120.40	115.30
Floor	0	97.75	114.75	128.62 ^a^	137.75	132.12	119.12
	Rosemary EO	96.60	114.20	127.40 ^a^	134.70	127.60	116.40
	Cinnamon EO	97.10	114.300	127.50 ^a^	136.80	128.60	117.90
SEM ^2^		0.825	0.840	0.713	0.728	0.769	0.547
Probability							
Housing system		<0.001	<0.001	<0.001	<0.001	<0.001	0.001
EOs		0.324	0.079	0.056	0.164	0.001	0.032
Housing × EOs		0.806	0.230	0.001	0.112	0.377	0.071

Means in the same column within each classification bearing different letters are significantly different. (*p* < 0.05 or 0.01).^1^ EOs: Essential oils, EO: Essential oil. ^2^ SEM: standard error mean.

**Table 6:** Feed conversion of laying hens as affected by different housing systems, essential oils and their interaction during the experiment.

Items		Feed Conversion (g Feed/g Egg) During					
		28–36 week	36–44 week	44–52 week	52–60 week	60–68 week	68–76 week
Housing system							
	Cage	2.23	2.39	2.60	2.75	2.85	3.08
	Floor	2.60	2.65	2.87	3.18	3.24	3.37
Essential oils (EOs) ^1^							
	0	2.67 ^a^	2.67 ^a^	2.84 ^a^	3.24 ^a^	3.36 ^a^	3.58 ^a^
	Rosemary EO	2.28 ^b^	2.40 ^b^	2.60 ^b^	2.79 ^b^	2.88 ^b^	3.04 ^b^
	Cinnamon EO	2.30 ^b^	2.49 ^b^	2.76 ^a^	2.87 ^b^	2.89 ^b^	3.07 ^b^
Housing × EOs							
Cage	0	2.48	2.52	2.61 ^b^	2.94 ^a^	3.13	3.37 ^b^
	Rosemary EO	2.12	2.30	2.54 ^c^	2.65 ^b^	2.76	3.00 ^cd^
	Cinnamon EO	2.09	2.36	2.65 ^bc^	2.66 ^b^	2.67	2.88 ^d^
Floor	0	2.86	2.82	3.06 ^a^	3.55 ^a^	3.59	3.79 ^a^
	Rosemary EO	2.44	2.50	2.67 ^b^	2.93 ^a^	3.01	3.07 ^c^
	Cinnamon EO	2.52	2.63	2.87 ^b^	3.07 ^a^	3.12	3.25 ^b^
SEM ^2^		0.035	0.046	0.055	0.058	0.056	0.058
Probability							
Housing system		<0.001	<0.001	<0.001	<0.001	<0.001	<0.001
EOs		<0.001	<0.001	0.002	<0.001	<0.001	<0.001
Housing × EOs		0.291	0.569	0.036	0.040	0.147	0.014

Means in the same column within each classification bearing different letters are significantly different. (*p* < 0.05 or 0.01). ^1^ EOs: Essential oils, EO: Essential oil. ^2^ SEM: standard error mean.

**Table 7:** Egg quality of laying hens as affected by different housing systems, essential oils and their interaction during the experiment.

Items		Shell Thickness (µm)	Eggshell %	Yolk %	Albumin %	Egg Index	Yolk Index	Haugh Unit
Housing system								
	Cage	0.36	8.79	28.80	62.39	77.34	22.54	82.53
	Floor	0.35	8.50	28.62	62.86	76.49	22.65	80.99
Essential oils (EOs) ^1^								
	0	0.35	8.54	28.49 ^b^	62.96 ^a^	75.56 ^c^	23.55 ^a^	79.55 ^c^
	Rosemary EO	0.35	8.70	28.90 ^a^	62.39 ^b^	77.34 ^b^	22.17 ^b^	82.40 ^a^
	Cinnamon EO	0.36	8.71	28.75 ^ab^	62.52 ^b^	77.84 ^a^	22.07 ^b^	83.33 ^a^
Housing × EOs								
Cage	0	0.36	8.77	28.56	62.66	76.10	23.33 ^a^	80.50
	Rosemary EO	0.37	8.83	29.07	62.09	77.84	22.29 ^b^	83.27
	Cinnamon EO	0.37	8.78	28.79	62.42	78.08	22.02 ^b^	83.82
Floor	0	0.35	8.30	28.41	63.27	75.03	23.78 ^a^	78.61
	Rosemary EO	0.34	8.56	28.73	62.70	76.84	22.05 ^b^	81.52
	Cinnamon EO	0.36	8.64	28.72	62.63	77.60	22.14^b^	82.85
SEM ^2^		0.091	0.801	0.110	0.134	0.177	0.121	0.416
Probability								
Housing system		0.025	<0.001	0.059	<0.001	<0.001	0.275	<0.001
EOs		0.480	0.099	0.007	0.002	<0.001	<0.001	<0.001
Housing × EOs		0.378	0.178	0.465	0.252	0.233	0.041	0.519

Means in the same column within each classification bearing different letters are significantly different (*p* < 0.05 or 0.01). ^1^ EOs: Essential oils, EO: Essential oil. ^2^ SEM: standard error mean.

**Table 8:** Blood picture of laying hens as affected by different housing systems, essential oils and their interaction during the experiment.

Items ^1^		WBC (× 10^3^/mm^3^)	RBC (× 10^6^/mm^3^)	PCV %	HB %	Eosino %	Lympho %	Hetero %	Baso %	Mono %
Housing system										
Cage		23.78	3.21	29.17	14.14	8.33	35.71	23.43	1.08	5.36
Floor		23.86	3.22	29.16	14.19	8.38	35.88	23.54	1.08	5.35
Essential oils (EOs) ^2^										
0		23.77	3.19	29.12	14.11	8.28	35.13 ^b^	23.38	1.05 ^b^	5.21 ^b^
Ros EO		23.93	3.22	29.08	14.22	8.34	35.93 ^ab^	23.63	1.10 ^a^	5.41 ^ab^
Cinn EO		23.76	3.22	29.29	14.17	8.44	36.33 ^a^	23.45	1.09 ^a^	5.46 ^a^
Housing × EOs										
Cage	0	23.77	3.22 ^a^	29.07	14.10	8.35	35.60	23.37	1.07 ^b^	5.30
	Ros EO	23.88	3.21 ^a^	29.16	14.24	8.26	35.58	23.50	1.08 ^b^	5.38
	Cinn EO	23.70	3.20 ^a^	29.28	14.08	8.38	35.96	23.42	1.08 ^b^	5.42
Floor	0	23.77	3.17 ^b^	29.17	14.12	8.22	34.67	23.40	1.02 ^b^	5.12
	Ros EO	23.99	3.23 ^a^	29.00	14.20	8.42	36.28	23.76	1.11 ^a^	5.44
	Cinn EO	23.81	3.25 ^a^	29.314	14.26	8.50	36.70	23.48	1.10 ^a^	5.50
SEM ^3^		0.108	0.016	0.145	0.078	0.085	0.364	0.140	0.013	0.088
Probability										
Housing system		0.377	0.432	0.932	0.437	0.479	0.585	0.293	0.966	0.853
EOs		0.173	0.222	0.237	0.422	0.236	0.018	0.178	0.006	0.018
Housing × EOs		0.840	0.029	0.605	0.360	0.260	0.075	0.624	0.030	0.260

Means in the same column within each classification bearing different letters are significantly different (*p* < 0.05 or 0.01). ^1^ WBC: White blood cells, RBC: Red blood cells, PCV: Packed cell volume, HB: Hemoglobin, Eosino: Eosinophils, Lympho: Lymphocytes, Heter: Heterophils, Baso: Basophils, Mono: Monocytes. ^2^ EOs: Essential oils, Ros EO: Rosemary essential oil, Cinn EO: Cinnamon essential oil. ^3^ SEM: Standard error mean.

**Table 9:** Immunity and antioxidant parameters of laying hens as affected by different housing systems, essential oils and their interaction during the experiment.

Items ^1^		Phagocytic Index	Phagocytic Activity	ND 60 W	AI H5 60 W	AI H9 60 W	MDA (nmoles/mL)	GPx (U/gHb)	SOD (U/gHb)
Housing system									
Cage		1.62	16.35	2.89	2.75	2.62	2.13	18.26	72.38
Floor		1.63	16.24	2.75	2.61	2.55	2.31	22.63	75.15
Essential oils (EOs) ^2^									
0		1.53	15.75	2.63 ^b^	2.36 ^b^	2.22 ^b^	2.50 ^a^	20.62	81.00 ^a^
Ros EO		1.66	16.45	2.86 ^a^	2.78 ^a^	2.68 ^a^	2.21 ^b^	20.80	73.70 ^b^
Cinn EO		1.69	16.70	2.97 ^a^	2.89 ^a^	2.86 ^a^	1.97 ^c^	20.00	66.60 ^c^
Housing × EOs									
Cage	0	1.57	16.37	2.82	2.49	2.33	2.45	15.00 ^c^	77.75
	Ros EO	1.62	16.20	2.86	2.86	2.68	2.10	20.60 ^b^	74.00
	Cinn EO	1.68	16.50	2.99	2.89	2.86	1.86	19.20 ^b^	65.40
Floor	0	1.50	15.12	2.44	2.23	2.12	2.55	26.25 ^a^	84.25
	Ros EO	1.70	16.70	2.86	2.71	2.68	2.32	21.00 ^b^	73.40
	Cinn EO	1.70	16.90	2.96	2.89	2.86	2.08	20.80 ^b^	67.80
SEM ^3^		0.072	0.47	0.095	0.085	0.081	0.070	0.882	2.15
Probability									
Housing system		0.892	0.752	0.070	0.072	0.304	0.007	<0.001	0.145
EOs		0.135	0.125	0.004	<0.001	<0.001	<0.001	0.644	<0.001
Housing × EOs		0.600	0.128	0.093	0.366	0.384	0.669	<0.001	0.318

Means in the same column within each classification bearing different letters are significantly different (*p* < 0.05 or 0.01). ^1^ ND: Newcastle disease, AI H5: Avian influenza H5, AI H9: Avian influenza H9, MDA: Malondialdehyde, GPX: Glutathione peroxidase, SOD: Superoxide dismutase. ^2^ EOs: Essential oils, Ros EO: Rosemary essential oil, Cinn EO: Cinnamon essential oil. ^3^ SEM: Standard error mean.

**Table 10:** Blood chemistry of laying hens as affected by different housing systems, essential oils and their interaction during the experiment.

Items		Cholesterol (mg/dl)	Protein (g/dl)	Calcium (m mol/L)	Phosphorus (m mol/L)	Urea (m mol/L)	Creatinine (m mol/L)	ALT ^1^ (U/L)	AST ^2^ (U/L)
Housing system									
Cage		190.43	3.49	2.71	2.21	5.31	0.44	20.40	86.10
Floor		193.56	3.50	2.69	2.21	5.54	2.65	21.08	89.41
Essential oils (EOs) ^3^									
0		206.50 ^a^	3.51	2.44 ^c^	2.08 ^c^	5.75 ^a^	0.51	22.62 ^a^	99.37 ^a^
Ros EO		187.30 ^b^	3.49	2.76 ^b^	2.22 ^b^	5.35 ^b^	3.78	20.40 ^b^	83.70 ^b^
Cinn EO		182.20 ^c^	3.48	2.89 ^a^	2.33 ^a^	5.18 ^b^	0.35	19.20 ^b^	80.20 ^c^
Housing × EOs									
Cage	0	204.50	3.54	2.43	2.09 ^c^	5.65	0.51	22.00	96.50
	Ros EO	185.80	3.47	2.74	2.19 ^b^	5.18	0.44	20.40	81.40
	Cinn EO	181.00	3.45	2.93	2.36 ^a^	5.10	0.37	18.80	80.40
	0	208.50	3.48	2.45	2.07 ^c^	5.85	0.50	23.25	102.25
Floor	Ros EO	188.80	3.51	2.77	2.26 ^a^	5.52	7.12	20.40	86.00
	Cinn EO	183.40	3.51	2.84	2.30 ^a^	5.26	0.33	19.60	80.00
SEM ^4^		1.830	0.093	0.031	0.023	0.065	2.86	0.53	1.28
Probability									
Housing system		0.057	0.883	0.636	0.797	<0.001	0.373	0.145	0.006
EOs		<0.001	0.966	<0.001	<0.001	<0.001	0.427	<0.001	<0.001
Housing × EOs		0.919	0.797	0.144	0.026	0.372	0.429	0.533	0.067

Means in the same column within each classification bearing different letters are significantly different (*p* < 0.05 or 0.01). ^1^ ALT: Alanine transferase. ^2^ AST; Aspartate transferase. ^3^ EOs: Essential oils, Ros EO: Rosemary essential oil, Cinn EO: Cinnamon essential oil. ^4^ SEM: Standard error mean.
